# Correction: Evaluating adherence to government recommendations for post-exposure rabies vaccine among animal-bite victims: A hospital-based study in Bangladesh

**DOI:** 10.1371/journal.pgph.0002909

**Published:** 2024-01-31

**Authors:** Sadia Tamanna, Dilruba Yasmin, Sumon Ghosh, M Mujibur Rahaman, Amit Kumar Dey, Tushar Kumar Das, Sukanta Chowdhury

## Notice of Republication

This article was republished on January 24^th^, 2024, to correct the author list and include M Mujibur Rahaman as the fourth author. Please download this article again to view the correct version. The originally published, uncorrected article and the republished, corrected articles are provided here for reference.

## Supporting information

S1 File Originally published, uncorrected article. (PDF)

S2 File Republished, corrected article. (PDF)

## References

[1] Tamanna S, Yasmin D, Ghosh S, Mujibur Rahaman M, Dey AK, Das TK, et al. (2023) Evaluating adherence to government recommendations for post-exposure rabies vaccine among animal-bite victims: A hospital-based study in Bangladesh. PLOS Glob Public Health 3(11): e0002506. 10.1371/journal.pgph.0002506 37963109 PMC10645289

