# Supplementary material for: Evaluating adherence to government recommendations for post-exposure rabies vaccine among animal-bite victims: A hospital-based study in Bangladesh
Source: PLOS Glob Public Health. 2023 Nov 14;3(11):e0002506. doi: 10.1371/journal.pgph.0002506 (PMC10645289; doi:10.1371/journal.pgph.0002506)
Supplement: Attachment — Submitted filename: PGPH-D-23-00281_Reviewers comments_responses.docx [file pgph.0002506.s002.docx]

**Reviewer 1:**

**Comment:** Consent is mentioned but ethics committee approval is not mentioned.

**Response:** Thanks for your comment. We have included the ethical statement in the revised manuscript (page no. 4, line no. 114-115).

**Comment:** The study time frame is Jan-Aug 2022. Did the pandemic have an impact on the follow up? This would become important.

**Response:** We appreciate about reviewer’s concern. Though our study has been performed between Jan-Aug, 2022, pandemic situation did not have any effect on people livelihood during the follow up. People are free to move everywhere without any restriction. Participants who did not complete the vaccination, showed different reason instead of pandemic situation that we mentioned in the manuscript. As a result, we did not mention the pandemic as a reason of incompletion or the delay of vaccination process in our data Table 4.

**Comment:** Rather than saying that the hospital follows "proper treatment" guidelines, it may be better to say " WHO guidelines for Post exposure prophylaxis were followed.

Response: Thanks for your suggestion. We have revised the line in our revised manuscript as per your suggestion (page no. 3, line no. 76-77).

**Comment:** It is not clear which regime was followed? Zagreb? Essen? IM or ID? All of this will impact adherence. 3 doses in one week or more?

**Response:** Thank you for your query. We apologize for not clearing the regimen of the rabies vaccination. At the first of our data collection, the nurses of our study hospital followed the Essen regimen where number of 4 IM doses were given to patients. After 2 months of our study period, the authority of our study site had decided to follow the Zagreb regimen and continued to provide 3 IM doses to patients. But the patients, who received 4 IM doses, were followed the Essen regimen. We have stated this situation in the result section of the revised manuscript (page no. 5, line no. 146-150).

**Comment:** Does the hospital give the vaccination and RIG free?

**Response:** Government provides vaccine and RIG free of cost to the victims but the patient had to pay only 0.09US$ in average to purchase a syringe. This information is mentioned in our manuscript also, though the cost of RIG was not mentioned. We have included additional information in the revised manuscript (page no. 5, line no. 167-168, 187-188).

**Comment:** Multiple independent variables have been collected but I do not see a regression analysis.

**Response:** Thank you for your comment. The main objective of this explorative study is to describe the adherence to government recommendations for post-exposure rabies vaccine among animal-bite cases. Over the study period, majority of the participants (n=352, 77%) were advised to take 3 - dose and 100% of them completed 3 - dose of vaccine. We did not find any compare group who did not complete 3 - dose of vaccine. Therefore, we did not perform any regression analysis to identify association between completeness of vaccination and factors.

**Comment:** Classification of wounds into Cat I, II, III would help.

**Response:** Thanks for your suggestion. We have added the classification of Cat I, II, III in the revised manuscript (page no. 2, line no. 58-60).

**Comment:** A lot of information in there in multiple tables. Some critical information needs to be in the text too.

**Response:** Thank you for your suggestion. We have added other important information in the revised manuscript (line numbers 130,143, 149, 152, 158-159, 223).

**Comment:** Range, SD missing for many variables- for example - age, time to wound washing.

**Response:** We agree with your comment. We we have mentioned the range of age and time to wound wash in the revised manuscript (line numbers 130, 152).

**Reviewer 2:**

**Comment:** Based on the reported state of the biting animal, 20% were considered suspect for rabies. It would be helpful to understand what criteria were used for this classification. For example, if self-reported by the patient or probed by the clinician for symptoms diagnostic for rabies?

**Response:** Thanks for sharing info and comment. In this study, we only selected animal bite victims who had a history of animal bites within the last 30 days and received first dose of rabies immunoglobulin (IG) or the rabies vaccine between January and August, 2022. So, we have not considered any suspect cases during participants selection.

**Comment:** 50% of biting animals were cats (perhaps not surprising in a setting like Dhaka), but were many cats also suspect for rabies (this is relevant because some places in Asia suggest increasing cat bites might suggest the emergence of a new reservoir)?

**Response:** We appreciate your query. Many study participants were exposed to cat scratch/bite. Our study could not define or exclude the role of cat as reservoir host. However, large number of cat’s bite/scratch is the matter of concern for rabies transmission to humans. Cats is considered as one of the susceptible host for rabies.

**Comment:** It would have been valuable to determine if the biting animal remained alive/healthy by day 7 (final vaccination), and thus the reliability of the health status of the animal reported (showed signs of rabies). Was this information collected?

**Response:** Thanks for your comment. The majority of the biting animals were apparently healthy (85%, n=388, 95% CI: 81-88%) during the day of bite/scratch/licking and 11 animals (2%) were died. The information was already included in the manuscript (line number 145-146 & Table 2).

**Comment:** I would be much more interested in adherence to PEP amongst this subgroup (i.e. where the animal is suspect rabid) versus all enrolled patients. Presumably this group will represent the majority of those truly at risk from rabies but protected by PEP. For those bitten by non-rabid animals, arguably poor compliance might be sensible if they know that the biting animal is healthy. It would be useful to consider risk when comparing compliance (in the first paragraph of the discussion).

**Response:** Thank you for your comment. We agree with you. Your propose approach certain generate strong evidence on adherence to PEP amongst high risk population. This approach require high resources and time. A community based study is needed to collect this information. Our study was hospital based and we did not collect this information. We have addressed this in the study limitation section (page no. 8, line number 240-241).

**Comment:** The interactions amongst the different risk variables (site of exposure, provision of RIG, state of the biting animal) and delay to 1st dose are potentially insightful but appear to have not been considered.

**Response:** Thanks for your comment. The main objective of this explorative study is to describe the adherence to government recommendations for post-exposure rabies vaccine among animal-bite cases. Over the study period, majority of the participants (n=352, 77%) were advised to take 3 - dose and 100% of them completed 3 - dose of vaccine. We did not find any compare group who did not complete 3 - dose of vaccine. Therefore, we did not perform any regression analysis to identify association between completeness of vaccination and factors.

**Comment:** >90% of rabies deaths are amongst bite victims that did not receive any PEP. The manuscript reports the demographics of patients who got PEP, and found them to differ from the demographics of patients who died of rabies. What does this say about health seeking from vulnerable groups? Are patients attending for PEP perhaps a somewhat different group?

**Response:** We agree with your statement. The burden of rabies has significantly reduced in Bangladesh because of the improvement of people's adherence to government-recommended PEP. There is a high level of literacy, an improved transportation system, higher income, easy access to hospitals, and an increase in awareness among people living in Dhaka that may account for the high level of PEP compliance.

**Comment:** Is it possible that the patient enrolment affected adherence? Would have been useful to compare with retrospective study where patients were matched for adherence.

**Response:** We conducted the study in Dhaka City where people had high level of literacy, an improved transportation system, higher income, easy access to hospitals, and an increase in awareness that may account for the high level of vaccination compliance.

**Comment:** The recommendation to reduce rabies exposures by avoiding stray dogs is naive. When a dog has rabies (including the many owned dogs that are also infected) it is actually quite hard to avoid being bitten. This may also explain the demographic of rabies deaths reported from Bangladesh!

**Response:** We agree with reviewer’s comment. We have mentioned in the manuscript (page number 7, line number 213-214, 238-239).

**Comment:** The barriers to bite victims receiving prompt PEP also appears very superficial. I would suggest a more considered and in-depth evaluation of why bite patients (specifically those at highest risk, bitten by likely rabid animals) delayed. And most importantly why rabies patients who did not receive PEP failed to do so.

**Response:** We agree with you. The limited resources did not allow us to perform in-depth exploration. Considering your suggestion, we have added a recommendation for further studies in the conclusion section of revised manuscript (page no. 8, line no. 253-254).

**Comment:** In several places I understood the authors to report PEP to comprise either vaccination or RIG. RIG without vaccine does NOT protect against rabies, so care should be taken to avoid conveying misleading information (similarly point about wound washing in 2nd paragraph of introduction).

**Response:** Thanks for your comment and we agree with you. We have revised introduction section considering your suggestion (page no. 2 line no. 49-54).

**Comment:** I think it would be helpful to reflect on how practices could be further improved: Although RIG is only required for severe exposures, 85% of patients received RIG, but <10% had bites to the face or neck. This disparity probably needs highlighting (I didn’t see if RIG is also provided free of charge but seems like it is being overprescribed).

**Response:** We appreciate reviewer’s comment. As per WHO recommendations, RIG with vaccines should be given if the exposure is category III. In our study, most of the cases were under category III, and so approximately 85% patients received RIG with vaccines. Vaccines and RIG were provided free of cost.

**Comment:** Cite primary studies for statistics/ reporting/ estimates; many of the references used do not relate to the point being made.

**Response:** Thanks for your suggestion. We have updated references considering your suggestion.

**Comment:** Avoid using the term ‘treatment’ for rabies. Sadly, there is no treatment for this disease.

**Response:** Thanks for your suggestions and we have revised manuscript accordingly.

**Comment:** Table 5. Is the total cost for managing the animal bite excluding lost working time? I would justify and clarify this otherwise the calculation looks incorrect.

**Response:** No cost due to loss of working time was not included during cost estimation. We have added this info in the revised manuscript (page no. 6 line no. 206).

**Comment:** Clean up references (see for example the bad formatting for reference 9). Data should be published with the paper! More generally, is adherence in Dhaka likely to be higher than in other more rural parts of the country? (and is rabies incidence also likely to be lower in Dhaka given that dog vaccination is carried out (semi?) regularly in the city?

**Response:** We have revised references. We submitted data during manuscript submission to the journal. We agree with you regarding the statement “adherence in Dhaka likely to be higher than in other more rural parts of the country. We have mentioned it in the limitation section.

**Reviewer 3:**

**Comment:** My opinion is that a better presentation and deeper analysis could improve the manuscript. For example, 5 tables are too much. You can use figures, graphs and tables. Analysis and data interpretation could be improved.

**Response:** Thank you for your suggestion. The main objective of this explorative study is to describe the adherence to government recommendations for post-exposure rabies vaccine among animal-bite cases. Over the study period, majority of the participants (n=352, 77%) were advised to take 3 - dose and 100% of them completed 3 - dose of vaccine. We did not find any compare group who did not complete 3 - dose of vaccine. Therefore, we did not perform deeper analysis such as multivariate regression to identify association between completeness of vaccination and factors.

**Comment:**

**Introduction**

- Introduction line 6: “is not or incorrectly administrated” instead of is “not administered or is administered incorrectly”.
- Introduction line 6 and 7: Improve the phrase “Over 59,000 people die from rabies each year around the world” to for example “According to WHO, 59000 human deaths per year are recorded globally”.
- Introduction lines 8-10: could you please improve the phrase “The incidence of rabies can be prevented or reduced by vaccination or immunoglobulin (IG) or immediate washing the wound or animal vaccination or animal birth control and also improving awareness on rabies”
- Introduction paragraph 3: “Bangladesh is placed third worldwide” instead of Bangladesh places third worldwide”

**Response:** Thank for your valuable suggestions and inputs. We have revised the mentioned lines of Introduction as per your suggestions (page no.2 line no.45-61).

**Comment: Method**

- “Methods” instead of “Method” (titer of Methods part)
- Methods line 1: avoid repetition (improve the title)

**Response:** We agree with your comments. We have revised the manuscript according to your comments for Methods (page no.3 line no.94).

**Comment: Result**

- Results, paragraph 1, line 1: “We enrolled a total of (or in total) 457 animal bite victims” instead of “We enrolled total 457 animal bite victims”.

**Response:** We appreciate your suggestion. We have changed the line as per your suggestion (page no.4 line no.152).

**Comment:** I recommend further revision for English and structure.

**Response:** Thank you for your correction. We have again checked the English, grammars and structures. After checking, we have revised the manuscript where the corrections were needed.
